# A new immunization and treatment strategy for mouse mammary tumor virus (MMTV) associated cancers

**DOI:** 10.18632/oncotarget.7762

**Published:** 2016-02-26

**Authors:** Ori Braitbard, Maayan Roniger, Allan Bar-Sinai, Dana Rajchman, Tamar Gross, Hillel Abramovitch, Marco La Ferla, Sara Franceschi, Francesca Lessi, Antonio Giuseppe Naccarato, Chiara M. Mazzanti, Generoso Bevilacqua, Jacob Hochman

**Affiliations:** ^1^ Department of Cell and Developmental Biology, Alexander Silberman Institute of Life Sciences, The Hebrew University of Jerusalem, Jerusalem, Israel; ^2^ FPS - Pisa Science Foundation, Pisa, Italy; ^3^ Department of Pathology, University of Pisa, Pisa, Italy

**Keywords:** signal peptide, mouse mammary tumor virus, breast cancer, vaccine, adoptive cell transfer, monoclonal antibodies, Pathology Section

## Abstract

Mouse Mammary Tumor Virus (MMTV) causes mammary carcinoma or lymphoma in mice. An increasing body of evidence in recent years supports its involvement also in human sporadic breast cancer. It is thus of importance to develop new strategies to impair the development, growth and metastasis of MMTV-associated cancers. The signal peptide of the envelope precursor protein of this virus: MMTV-p14 (p14) is an excellent target for such strategies, due to unique characteristics distinct from its regular endoplasmic reticulum targeting function. These include cell surface expression in: murine cancer cells that harbor the virus, human breast cancer (MCF-7) cells that ectopically express p14, as well as cultured human cells derived from an invasive ductal breast carcinoma positive for MMTV sequences. These findings support its use in signal peptide-based immune targeting. Indeed, priming and boosting mice with p14 elicits a specific anti-signal peptide immune response sufficient for protective vaccination against MMTV-associated tumors. Furthermore, passive immunization using a combination of anti-p14 monoclonal antibodies or the transfer of T-cells from immunized mice (Adoptive Cell Transfer) is also therapeutically effective. With reports demonstrating involvement of MMTV in human breast cancer, we propose the immune-mediated targeting of p14 as a strategy for prevention, treatment and diagnosis of MMTV-associated cancers.

## INTRODUCTION

Mouse Mammary Tumor Virus (MMTV) is a type B retrovirus known to cause mammary carcinoma or lymphoma in mice [1] [2], [3]. An increasing body of evidence in recent years supports the notion that this virus is involved in over 30% of patients with sporadic breast cancer [4, 5] (and references within). This may be related to both geographical distribution [6] and type of the disease, as it was previously reported that, in Tunisian and American women with inflammatory breast cancer, a larger percentage (up to 70%) of cases demonstrated MMTV sequences [7]. It was also reported that cells isolated from ascites or pleural effusions of patients with metastatic breast cancer contained MMTV sequences in their DNA, expressed the MMTV Env protein and showed, by electron microscopy, retroviral particles similar to the mouse virus. The same group reported detection of HMTV (Human Mammary Tumor Virus) proteins in human breast cancer cells 90%-98% homologous to MMTV. [8, 9]. Whether the virus plays a role in the pathogenesis of the disease or whether, for example, women that contract the disease are prone to viral infection, is not yet established. It is noteworthy, however, that the virus was shown to infect human breast cells and propagate *in vitro* [10, 11]. Recently, saliva was proposed as a route for inter-human infection by MMTV [12]. Recent reviews summarized the current knowledge [13] stressing the significance of continuing research in this field [14]. In addition, a human betaretrovirus (HBRV) bearing 91-99% identity to MMTV has been linked also with primary biliary cirrhosis [15] and frequently observed at the site of disease as well as in biliary epithelia of patients with autoimmune hepatitis and cryptogenic liver disease [16]. Here, too, it is not established whether the virus is causally linked to the development of liver disease or whether it represents an epiphenomenon.

Signal peptides are N-terminal extensions on nascent secretory and membrane proteins (typically including 15-25 amino acid residues) that mediate insertion into, or translocation across the membrane of the endoplasmic reticulum (ER). Usually, once their targeting function is completed, signal peptides are degraded by signal peptide peptidase. However, a growing number of signal peptides have been shown to carry out additional (post-ER targeting) functions. For example, the signal peptides of several arenaviral glycoproteins (Lassa, Junin, and lymphocytic choriomeningitis virus) remain membrane-inserted. They are necessary for processing of the mature glycoprotein complexes, and important for viral infection [17-21]. In hepatitis C virus poly-protein, signal peptide peptidase processing results in the release of the core protein into the cytosol [22] and is essential for HCV assembly [23] [24]. In the case of the HLA-A*0301 molecule, fragments derived from the signal peptide are presented at the cell surface and monitor the expression of their corresponding protein for immune surveillance by NK cells [25].

Previously, we demonstrated that the signal peptide of the envelope precursor protein of MMTV, after fulfilling its ER targeting function, is localized to nucleoli of cells that harbor the virus (murine mammary carcinoma and lymphoma) [26], [27] [28], as well as to nucleoli of a number of human breast cancer cases [29]. The nucleolar localization of this unusually long signal peptide (98 amino acids) named by us MMTV-p14, or p14 for short (according to its electrophoretic mobility), is not unique to MMTV. It was subsequently demonstrated that the signal peptide of another beta retrovirus: HERV-K(HML-2), associated with testicular germ cell tumors, encodes a 13kDa signal peptide that also translocates to nucleoli [30]. p14 was initially identified using a monoclonal antibody (M-66) belonging to a class of antibodies directed against cell surface epitopes of immunogenic murine lymphoma cell variants that harbor MMTV [31]. The epitope recognized by antibody M-66 was mapped (using competition and deletion analyses) to include the region of a functional nuclear localization signal [27]. p14 binds a number of target proteins, among them the nucleolar proteins B23 (Nucleophosmin) and ribosomal protein L5 (RPL5) [32]. The latter, as well as ErbB4, are also transcriptionally regulated by p14 [32]. Subsequent to our initial findings [26] [27], it was demonstrated that this signal peptide plays a key role (analogous to HIV-Rev) as nuclear export factor for intron containing viral transcripts [33] [34], thus defining MMTV as a complex virus. Recently, we reported that p14 is a phosphoprotein tumor modulator, endogenously phosphorylated by two serine kinases: CK2 at serine 65 and PKC at serine18. When mutated in the PKC phosphorylation site, p14 will function as an oncogene, while when mutated in the CK2 site it will function as an anti-oncogene. [32].

In view of these findings, the proposed association of MMTV with breast cancer, and its frequent presence in primary biliary cirrhosis [16], we investigated whether p14 can be used in the capacity of a tumor associated antigen. Here we report that p14 (or peptides thereof) is expressed on the cell surface of both murine and human cells that contain the virus or viral sequences. Since p14 is immunogenic, it serves as target for preventive vaccination against malignant cells that harbor MMTV, as well as a source for specific T-cells and monoclonal antibodies used in passive immunization, further demonstrating the multi-faceted nature and potential of this unique signal peptide.

## RESULTS

### P14 is expressed also on the surface of MMTV- associated cells

**Figure 1:**
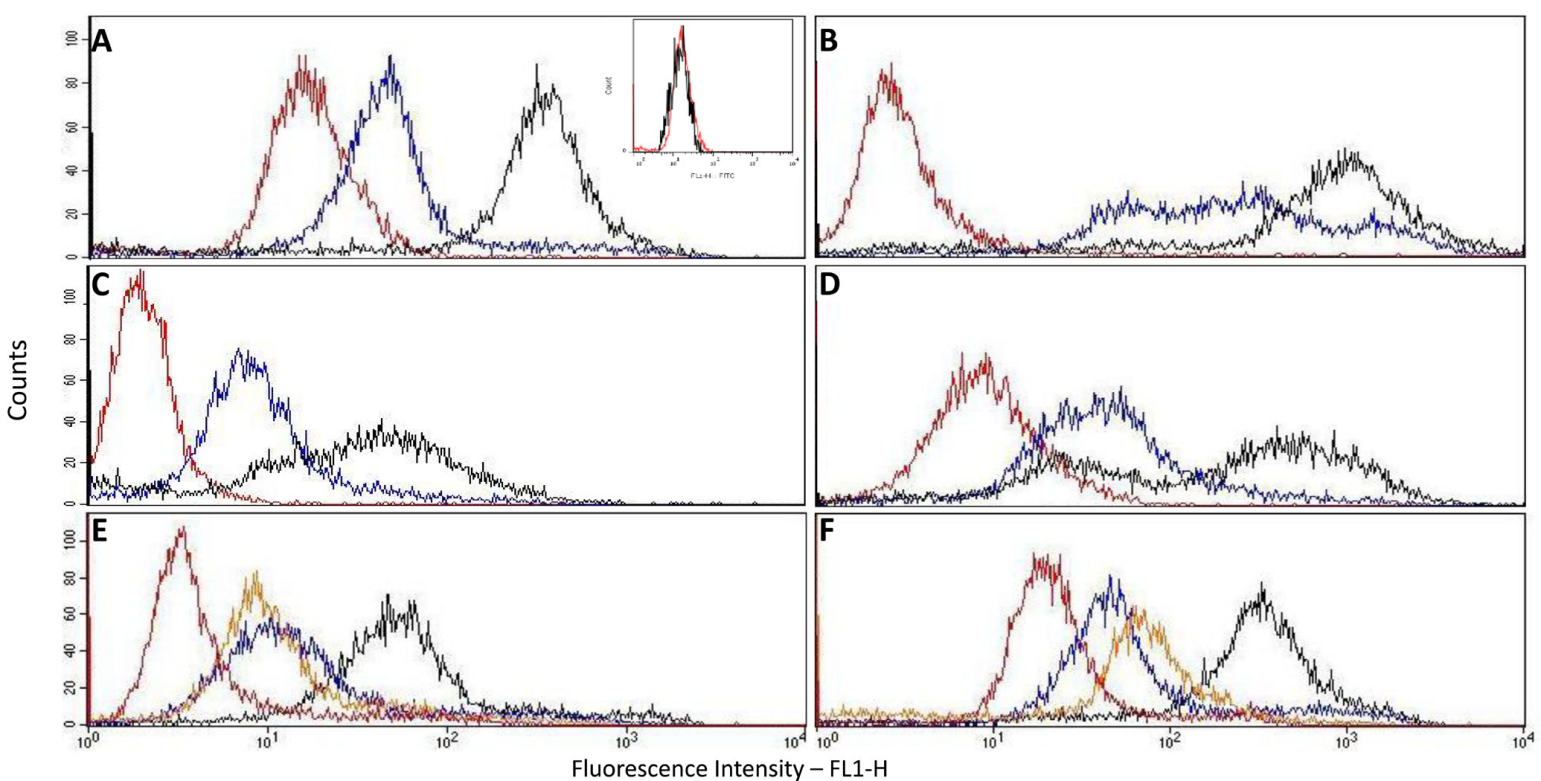
Cell surface and whole cell expression of p14 using flow cytometry In all samples: Red – intact cells (Control – no 1^st^ antibody); Blue – Intact cells (cell surface expression of p14); Black – Permeabilized cells (whole cell expression of p14); Orange – Permeabilized cells (Control – no 1^st^ antibody). Antibodies used: **A**.-**D**. &. **F**. - Rabbit polyclonal; **E**. – Mouse monoclonal M-66. Cells used: A – MCF-7 human breast cancer cells ectopically expressing p14 (insert – MCF-7 cells lacking p14: intact *vs.* control – no 1^st^ antibody); B-D – murine lymphoma T-67, mammary carcinoma 4T1 and mammary carcinoma Mm5MT, respectively, that harbor MMTV; E & F – MCF-7 cells expressing p14.

p14 is present not only inside cells as already shown, but also on the cell surface as demonstrated here by Flow Cytometry analysis of intact cells (Figure 1A-1D). Cell lines analyzed were: Human MCF-7 breast cancer cells that ectopically express p14, and the following MMTV-associated cells : Murine T-67 T-cell lymphoma, 4T1 and Mm5MT mammary carcinomas, all expressing endogenous p14. MCF-7 cells that do not express p14 were used as negative controls (Figure 1A insert). All p14 positive cells (Figure 1A-1D) show a clear increase of fluorescence intensity of labeled intact cells above their internal controls (no anti-p14 antibody). Moreover, permeabilized cells show an even higher level of fluorescence intensity, demonstrating that p14 is present also intracellularly.

Comparing Mab-M-66 with polyclonal anti-p14, as to their interaction with MCF-7 cells ectopically expressing p14, demonstrates (Figure 1E, 1F) that the epitope recognized by Mab-M-66 is exposed to the extracellular environment and that in both samples roughly 5-7% of the total p14 content is cell surface related (see Materials and Methods).

### Detection of p14 in human breast cancer cells expressing MMTV (−env related sequences)

**Figure 2:**
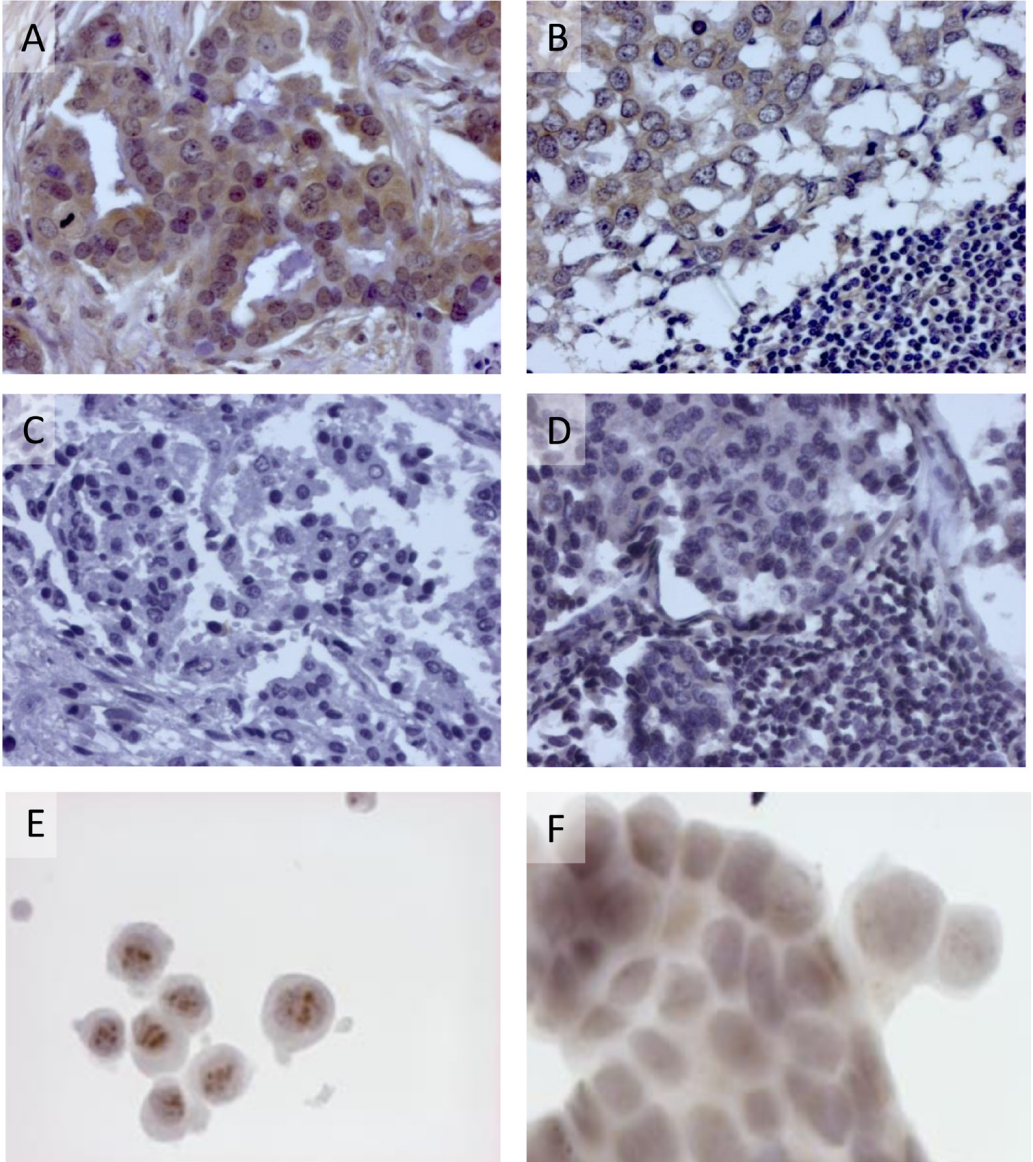
Expression of p14 in human breast cancer cells Detection was carried out using rabbit polyclonal anti-p14 and peroxidase staining. **A.** & **B.** - Invasive ductal carcinoma 2531 and lymph node metastasis previously reported positive for MMTV sequences, using PCR. **C.** & **D.** - Invasive ductal carcinoma (4018) and lymph node metastasis, previously reported negative for MMTV sequences, using PCR. **E.** Nucleolar detection of p14 in pBC cells derived from invasive ductal carcinoma 2531 that contain MMTV sequences, but not in **F.** control MCF-7 cells devoid of MMTV.

To determine the relevance of the above findings to human breast cancer, we tested whether human breast cancer cells, previously reported to contain MMTV-env sequences (using PCR),[4] express p14 at the protein level and whether p14 is also presented in these cells at the cell surface, as is the case in murine cells. Thus, an invasive ductal breast carcinoma (2531) positive for MMTV-related sequences demonstrates strong immune reactivity toward anti-p14 polyclonal antibody (using peroxidase labeling), both in the primary lesion (Figure 2A) as well as in a metastatic lymph node (Figure 2B). An invasive ductal breast carcinoma (4018), devoid of MMTV sequences, demonstrates no immune reactivity, in either the primary lesion (Figure 2C) or in a metastatic lymph node (Figure 2D). Furthermore, a primary cell line - pBC, derived from the positive infiltrating ductal carcinoma above. demonstrates immune reactivity, including nucleolar localization characteristic of p14 (Figure 2E). Control MCF-7 cells devoid of p14 show no such localization (Figure 2F) (see also Figure 1A insert, and [32]. The correlation observed between the PCR and protein analyses here, is strengthened by our recent findings [8] demonstrating that human salivary glands containing MMTV-env like sequences (using PCR) were also positive for p14 using immunhistochemistry. Negative salivary glands (using PCR) were also negative using immunohistochemistry.

**Figure 3:**
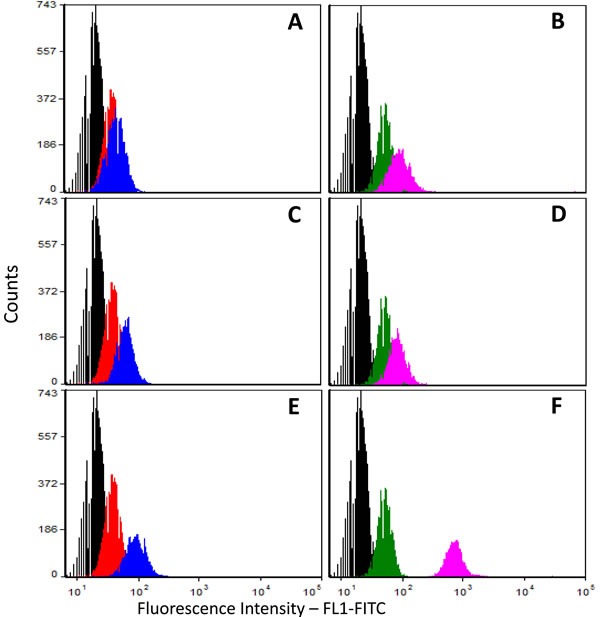
Cell surface and total cell expression of p14 in human breast cancer (pBC) cells Cells were subjected to flow cytometry using 3 different anti-p14 antibodies (mouse monoclonals M-66 and M-202, and mouse polyclonal). **A.**,**C.**& **E.** Non-permeabilized cells (cell surface expression); **B.**,**D.**& **F.** Permeabilized cells (total cell expression); Anti-p14 antibodies used: **A.** & **B.** Monoclonal M-66; **C.**& **D.** Monoclonal M-202; **E.** & **F.** Mouse polyclonal anti-p14. Histograms: Black - Unlabeled (control) cells; Red & Green - Cells labeled only with secondary antibody (Goat anti-mouse IgG1-FITC); Blue & Purple - Cells labeled with primary (polyclonal mouse anti-p14) antibody and secondary Goat anti-mouse IgG1-FITC-labeled antibody.

Next, we applied flow cytometry to the pBC cell line using three different anti-p14 antibodies: Mab M-66, a newly derived IgG1 Mab M-202, directed against a different p14 epitope, and a mouse polyclonal anti-p14 antibody. Figure 3A-3F demonstrates that the three antibodies recognize p14 in pBC cells, both on the cell surface and inside the cells. The above findings strongly suggest that at least two different epitopes of p14 (M-66 and M-202) are expressed on the cell surface of both murine cells that harbor MMTV and human breast cancer cells that contain MMTV-related sequences, as well as human breast cancer (MCF-7) cells that ectopically express p14. The enhanced signals generated with polyclonal anti-p14 antibodies over monoclonal antibodies are consistent with the cell surface expression of additional p14 epitopes. A similar conclusion is drawn from the findings in Figure 1E, 1F.

### Using p14 expressed on the cell surface as target for immune surveillance

**Figure 4:**
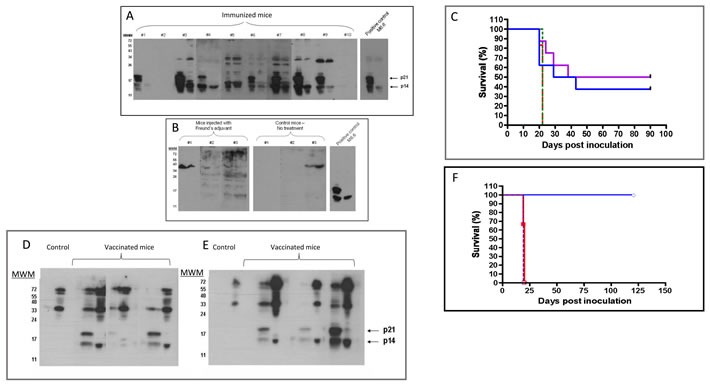
MMTV-p14 promotes the generation of anti-p14 antibodies and vaccinates Balb/C mice against T-67 lymphoma cells that contain MMTV **A.** - Sera (1/40 dilution) from individual mice (*n* = 10), primed and boosted with p14 in complete Freund's adjuvant (see materials and methods) were tested against extracts of T-67 (left lane of each sample), and T-25-Adh (right lane of each sample) lymphoma cells, using western blotting. Positive control- Monoclonal antibody M-66 (1/200 dilution). In addition to p14, T-67 cells express p21 which includes the p14 sequence extended into gp52. **B.** - Negative controls: Sera (1/40 dilution) from mice injected only with adjuvant and from naïve (non-inoculated) mice. **C.** - Mice were primed and boosted with p14 in complete Freund's adjuvant (see materials and methods) and subsequently challenged IP with 5×10^6^ (Violet line) (*N* = 8) or 10×10^6^ (Blue) (*N* = 8) T-67 cells. Control - Naïve mice challenged with 5×10^6^ (Dotted Red line) (*N* = 6) and 10×10^6^ (Dashed Green line) (*N* = 6) T-67 cells. **D.** & **E.** - Serum antibodies in individual mice after vaccination with p14 and a challenge with T-67 lymphoma cells. Individual sera (from 3 mice) were obtained 24 days after challenge with 5×10^6^ cells **D.**, and 10×10^6^ **E.** T-67 cells. Dilution of sera - 1/50 Sera were tested against extracts of T-67 (left lane of each sample) and T-25-Adh (right lane of each sample) cells as above. Note: Control sera (from naïve mice inoculated with T-67 cells) are devoid of anti-p14/21 antibodies. **F**. - Adoptive cell transfer from vaccinated mice protects naïve mice against T-67 lymphoma cells.6×10^7^ spleen cells from vaccinated mice (Blue line) that survived the challenge with T-67 cells were transferred IP into naïve mice (*N* = 4) followed by inoculation with 5×10^6^ T-67 cells per mouse. Control (Red line): Naïve mice (*N* = 4) inoculated with 6×10^7^ spleen cells from naïve mice followed by inoculation with 5×10^6^ T-67 cells per mouse. Control (Green line): Naïve mice (*N* = 4) inoculated with 5×10^6^ T-67 cells per mouse.

Recombinant p14 in complete Freund's adjuvant was used to prime and boost Balb/C mice. Four weeks after the first boost, the sera contained antibodies reacting with p14 in lysates of both T-67 and T-25-Adh lymphoma cells that harbor MMTV (Figure 4A). These antibodies also recognize p21 (p14 plus an extension into MMTV-gp52), which is specific to T-67 cells [35]. Sera of naïve mice (Figure 4B), as well as of mice injected with adjuvant alone, were devoid of anti-p14 (or p21) antibodies. Furthermore, when immunized mice were subsequently challenged with tumorigenic syngeneic T-67 cells (3 weeks after the final p14 boost), 50% (4/8) and 37% (3/8) of mice injected with 5 X10^6^ or 10 X10^6^ cells, respectively, did not develop tumors for at least 90 days post challenge (Figure 4C). Note that the profile of the proteins recognized by sera of the T-67 challenged individual mice changed, and additional, higher molecular weight antigens are also recognized (Figure 4D and 4E *vs*. Figure 4A). This is consistent with additional T-67 antigens playing a role in the immune response (see below).

When control mice, injected with PBS, were subjected to a challenge with T-67 cells, none of them survived (Figure 4C). Also, mice injected with adjuvant alone or p14 alone did not survive the challenge (not shown). Some of the vaccinated mice that did develop tumors showed longer survival periods (Figure 4C), suggesting a partial protection. These findings demonstrate the active immunization (preventive vaccination) potential of p14.

To test whether immune protection can be adoptively transferred, spleen cells from p14-vaccinated mice that survived the T-67 challenge, were transferred to naïve mice (IP inoculation of 6 × 10^7^ cells per mouse). This was followed by IP inoculation of 5×10^6^ T-67 cells per mouse. Treated mice (*N* = 4) survived the challenge with aggressive T-67 cells (Figure 4F). Neither naïve mice (*N* = 4), nor mice injected with cells (6 × 10^7^) prepared from the spleens of naive mice (*N* = 4) survived the challenge with T-67 cells (Figure 4F).

### Using p14 for protective vaccination (with alum as adjuvant)

**Figure 5:**
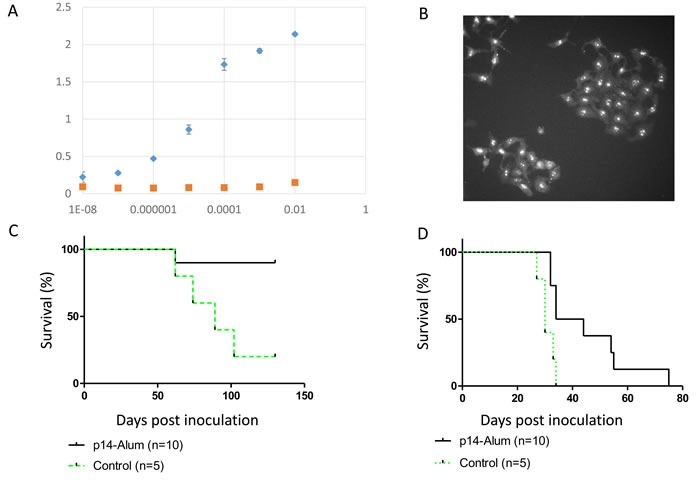
Protective vaccination using p14 in Alum-based adjuvant Mice were primed and boosted with p14 in Alum-based adjuvant (see materials and methods) and subsequently challenged with T-67 or 4T1 cells. **A.** & **B.** - Pooled sera prior to challenge with cells contain anti-p14 antibodies as validated by ELISA (see Materials and Methods) against decreasing dilutions of purified p14, and immune fluorescence (nucleolar localization) in MCF-7 cells ectopically expressing p14. Diamonds - serum from mice injected with p14 in Alum; Squares - Serum from mice injected with Alum only. **C.** - Survival of vaccinated mice (*N* = 10) challenged with 2×10^6^ T-67 lymphoma cells (Solid line). Control - Naïve mice (*N* = 5) challenged with 2×10^6^ T-67 cells (Dashed line). **D.**- Survival of vaccinated mice (*N* = 10) challenged with 1.5×10^3^ 4T1 mammary carcinoma cells (Solid line). Control - Naïve mice (*N* = 5) challenged with 1.5×10^3^ 4T1 cells (Dashed line).

We tested the efficacy of priming and boosting with p14 in Alum-based adjuvant ( which has been used in vaccination for over 70 years with an excellent safety record [36] on the subsequent *in vivo* development of two cell lines which harbor MMTV: T-67 lymphoma and murine 4T1 mammary carcinoma (an aggressive, poorly immunogenic and highly metastatic cell line) [37]. Pooled sera of mice primed and boosted with p14-Alum contained anti-p14 antibodies as demonstrated by ELISA (Figure 5A) and nucleolar localization (using immune fluorescence) of p14 in MCF-7 cells that ectopically express p14 (Figure 5B). The Alum-based vaccination protocol is effective in enhancing immune responsiveness, giving about 90% protection in the case of T-67 cells (Figure 5C) and extending survival in the case of 4T1 cells (Figure 5D).

### Immune-therapy using adoptive cell transfer

**Figure 6:**
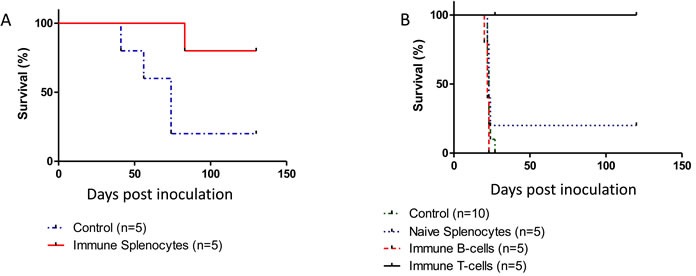
An immune response elicited solely against p14 is sufficient for protection against T-67 cells **A.** - Spleen cells (6×10^7^) from mice (*N* = 5) previously primed and boosted with purified p14 (in Alum-based adjuvant) were transferred into naïve mice followed by inoculation of 1.5×10^6^ T-67 cells (Solid line). Control: Spleen cells (6×10^7^) from naïve mice (*N* = 5) were transferred into naïve mice followed by inoculation of 1.5×10^6^ T-67 cells (Dashed line). **B.** - T-cells directed against p14 impair tumor development *in vivo*. Mice were inoculated with 2×10^6^ T-67 lymphoma cells followed by a single inoculation of 12×10^6^ T-cells (Solid, Black line) or 8×10^6^ B-cells (Dashed, Red line) negatively selected (see materials and methods) from spleens of mice primed and boosted with p14. Controls included mice inoculated only with T-67 (2×10^6^) cells (Dotted, Blue line) and mice inoculated with T-67 (2×10^6^) cells followed by inoculation of 15×10^6^ splenocytes from naïve mice (Dotted, Green line).

Mice were primed and boosted with purified p14 (in Alum-based adjuvant) but were not challenged with T-67 cells. Instead, transfer of spleen cells from these immunized mice into naïve mice (adoptive cell transfer) was carried out, followed by a challenge of the recipient mice with T-67 cells. As can be seen (Figure 6A), this adoptive transfer protocol is essentially as protective as in the above experiments, consistent with the notion that p14 (and immune effector cells specifically directed towards p14 epitopes) are sufficient for preventive vaccination against tumors that contain MMTV. Furthermore, adoptive T-cell transfer carried out with enriched T-cells (derived from spleens of mice immunized with p14 in Alum) was also highly protective against MMTV-associated T-67 tumors (Figure 6B).

### Immune-therapy using monoclonal anti-p14 antibodies

**Figure 7:**
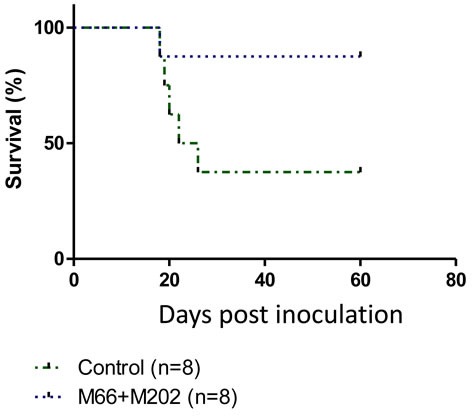
Combining two anti-p14 Mabs protects mice against T-67 lymphoma cells Mice (*N* = 8) were inoculated with 1.5×10^6^ T-67 lymphoma cells (Dotted line) followed by injections of Mabs M-66 + M-202 (25μg each) every other day for 4 weeks. Controls (Dashed line) included mice inoculated only with T-67 (1.5×10^6^) cells (*N* = 8).

Mabs M-66 and M-202 were injected separately (IP) into mice inoculated with T-67 cells. None of the mice survived longer than control mice inoculated only with T-67 cells (not shown). However, a combined injection of M-66 and M-202 into mice challenged with T-67 cells significantly enhanced their survival over control mice (Figure 7). These findings demonstrate that monoclonal antibodies directed against p14 can be applied for passive immunization against MMTV-associated tumors.

## DISCUSSION

Various post ER targeting functions of viral signal peptides (SPs) have been characterized in recent years, supporting the emerging concept of bioactive SPs as a retroviral strategy to modulate their host cell environment. This also includes modulation of the host immune response, as in the case of Human Cytomegalovirus (HCMV), where the signal peptide (through regulation of cell surface expression of two NK cell ligands) differentially affected two distinct NK cell evasion pathways [38]. A recent publication [39], reported that an intact signal peptide on dengue virus E protein (ER-targeted E protein) enhanced the immunogenicity for CD8^+^ T cells and generated superior antibody responses when expressed from the immunogenic recombinant vaccine vector Vaccinia Ankara.

The present study demonstrates that the MMTV signal peptide (p14) is expressed on the surface of MMTV-associated murine and human cells. Based on these findings it is also demonstrated that p14 can be applied for protective vaccination, having preventive efficacy towards aggressive murine tumors that harbor the parental (MMTV) virus. This immune reactivity can be adoptively transferred into naïve mice using purified T cells, thus further allowing selection of specific p14-targeting CTL clones. Furthermore, this study also demonstrates that monoclonal antibodies directed against p14 can be applied for passive immunization. Based on the reports of different groups regarding the association of MMTV with human breast cancer and primary biliary cirrhosis, p14 is a potential target for preventive vaccination, as well as for cytotoxic antibodies directed against such malignancies. Indeed, the cell surface expression of p14 on a human breast cancer cell line (pBC), as shown above, is a first indication that p14 might be used in such a capacity. Pertinent to this study are also our recent findings [12] that salivary glands (3/37) positive for MMTV (by PCR) were also positive for p14 by immune histochemistry (IHC) analysis with polyclonal anti-p14 antibodies. Salivary glands negative by PCR were also negative by IHC. The fact that the p14 sequence is unique, with no similarities to mammalian sequences, is of significance to this end. In fact, blast (NCBI Blast+) of the amino acid sequence of p14 *vs*. the uniprotkb-mammals database did not reveal any mammalian sequence identity longer than 4 amino acids. Furthermore, the sequence identity between the mouse p14 and that reported for a human retroviral homolog, is 86% [29].

The advantages of using p14 are its diverse characteristics and cellular locations making it a significant target for signal peptide-mediated therapeutics. Its therapeutic potential is independent of whether the virus plays a role in the pathogenesis of the disease or whether it is just a marker for specific cancer cells. Indeed, the intracellular (post-ER targeting) functions, cell surface expression and immunogenicity of p14, suggest a number of potential strategies for the targeting and treatment of MMTV-associated cancers in a clinical setting. These could be applied separately or in combination and may include: A) Prophylactic and therapeutic vaccination (active immunization) protocols using p14 or mutant p14s; B) The isolation of anti-p14 specific CTL clones (from primary tumor sites or metastatic lesions) directed against cell surface epitopes of MMTV-associated tumors; C) Monoclonal (humanized) anti-p14 antibodies as direct cytotoxic agents (passive immunization) or as vehicles for targeted drug delivery, including genetically engineered immunotoxins or chemically coupled conjugates with chemotherapeutic drugs; D) Targeting and intracellular introduction of anti-p14 monoclonal antibodies into MMTV-associated cancer cells (to impair affected cell growth) using immune conjugates between a p14 monoclonal and a translocator protein (like HIV-Tat). A similar approach (monoclonal antibody-HIV-Tat conjugate) was employed by us recently to impair the activity of the ABCB1 multi-drug transporter from within drug resistant cells, thus rendering them sensitive to chemotherapy [40]; E) Intracellular introduction of small molecule inhibitors of p14 based on molecular modeling and X-ray crystallography and the role of various mutations along its sequence. For example, based on our recent findings that a point mutation in the CK2 phosphorylation site of p14 results in tumor growth inhibition [32], small molecule inhibitors of CK2 could end up having a similar inhibitory effect in MMTV-associated tumors. By the same token, since a point mutation in the PKC phosphorylation site of p14 enhances tumorigenicity [32], activators of PKC could impair tumor growth. While this seems controversial in view of the long-time accepted dogma of PKC as a tumor promoter, recent work suggests PKC to function rather as a tumor suppressor [41]. F) Finally, there is the potential application of p14 (and mutant p14) as well as anti-p14 antibodies assays for (early?) diagnosis of MMTV-associated tumors. To that effect it is noteworthy that in a preliminary experiment, p14 was detected (using ELISA) in sera of mice inoculated with T-67 lymphoma and 4T1 mammary carcinoma cells and not in sera of naïve mice (Braitbard O, unpublished). Additional studies are needed to address these exciting possibilities.

While p14 is unique functionally, and unusually long for a signal peptide (98 amino acid residues), it is not an “orphan” signal peptide with regards to nucleolar localization [30]. It is therefore proposed that additional signal peptides derived from virally induced, or associated cancers, might be useful as targets for vaccination and other signal peptide-based therapies of such malignancies, further unraveling the secret lives of these intriguing multi-functional peptides.

## MATERIALS AND METHODS

### Cell culture

Cell culture materials were from Invitrogen (Carlsbad, CA), unless otherwise noted. Tumorigenic T-67 and non-tumorigenic T-25-Adh lymphoma cells (both containing MMTV) were derived from the T-25 murine lymphoma through continuous *in vivo* passages and *in vitro* selection, respectively [29]. Cells were grown in culture as previously described [35] Parental T-25 cells were derived from the S49 T-cell lymphoma (containing MMTV) induced in a Balb/C mouse by mineral oil [42]

MCF-7 human breast carcinoma cells, devoid of p14, or to which only p14 was introduced and stably expressed [43] [32], as well as Mm5MT (ATCC CRL-1637) [44] and 4T1 (ATCC CRL-2539) [45] Balb/C-derived mammary carcinoma cell lines (American Type Culture Collection, Rockville, MD), both expressing MMTV, were grown under conditions identical to the lymphoma cells, except with 10% fetal calf serum replacing horse serum.

All material of human origin was appropriately anonymized according to the guidelines established by the ethical committee of the University Hospital of Pisa

### Immunohistochemistry

Immunohistochemical assays were performed on formalin-fixed paraffin-embedded sections.as previously described [12] using rabbit polyclonal anti-p14 as primary antibody (at 1:500 dilution).

### Primary breast cancer (pBC) cells

Cell culture materials were from Lonza, (Basel, CH) unless otherwise specified.

The pBC cell line was generated from a specimen of human invasive ductal breast carcinoma, derived by the surgeons of the Breast Unit at the University Hospital of Pisa. This breast carcinoma was previously shown to contain MMTV-env sequences using nested PCR [4]. The specimen was dissected under sterile conditions by an experienced pathologist and only tumoral pieces were selected. Tissue fragments were carefully minced into 2-3 mm cubes in a small volume of growth medium and washed with a mixture of PBS (Phosphate Buffered Saline), penicillin/streptomycin and fungizone.

The tissue fragments were incubated overnight at 37°C under 5% CO_2_ with DMEM F12, 10% FBS (Fetal Bovine Serum) and 10 mg collagenase III (Invitrogen, Life Technologies). On the following day, the tissue fragments were centrifuged at 1200 rpm for 5 minutes and plated in a T25 flask with growth medium containing: DMEM F12, 1% penicillin/streptomycin, 1% hydrocortisone (Sigma), 1% fungizone, 5% FBS, 20 ng/ml hEGF (Human Epidermal Growth Factor, Miltenyi Biotec), 2% B27, 25ul Coleric Toxin (Reagent Proteins), 2ml Bovine Pituitary Extract (Invitrogen, Life Technologies).

### Mice

Balb/c female mice (6 - 8 weeks old) were obtained from Harlan (Israel) and held in a Specific pathogen free (SPF) facility, accreditation #1285 by AAALAC (Association for Assessment and Accreditation of Laboratory Animal Care). Mice were treated in accordance with NIH guidelines and approval by the institutional committee for ethics in animal experimentation.

### Western blotting

Was carried out as previously described [29].

### Cell surface and whole cell expression of p14 using flow cytometry

Cells fixed in 4 % paraformaldehyde were incubated with 1^st^ anti-p14 antibody, followed by FITC-conjugated 2^nd^ antibody (Santa Cruz Biotechnology, Santa Cruz, CA). Analysis was performed on an Excalibur Fluorescence Activated Cell Sorter (BD biosciences, San Jose, CA). Cellular permeabilization, where required, was performed by incubation in 0.1% Triton X-100 subsequent to fixation and prior to incubation with the 1^st^ anti-p14 antibody. Cell surface (intact cells) expression relative to whole cell (permeabilized cells) expression of p14 was calculated according to the following equation: (Intact cell median- Intact cell median control/(Permeabilized cell Median - Permeabilized cell median control) = % p14 on the cell surface. This is just a rough estimate as different epitopes may be differentially exposed to the external environment.

A similar protocol was used in the case of pBC cells, except that samples were analyzed on a CyFlow^®^ Cube 8 Sorter Flow Cytometer (Partec) using FCS express 4 software (De Novo Software™). At least 10,000 events per sample were collected.

### Immunizing mice with p-14

Recombinant p14-His (100 μg/0.2ml PBS) was used to prime and boost Balb/C mice: priming in complete Freund's adjuvant and 3 consecutive monthly boosts in incomplete Freund's adjuvant, subcutaneously. Alternatively, mice were primed and boosted intramuscularly with p14 using Alum (Sigma) as adjuvant. Mice were subsequently challenged intraperitoneally (IP) with T-67 lymphoma cells or into mammary fat pads with 4T1 mouse mammary carcinoma cells. Bleeding from the tail vein was used to monitor antibody presence in sera.

### ELISA

A standard protocol for ELISA (using peroxidase-conjugated secondary antibody) was used for detection of anti-p14 antibodies in pooled sera from mice immunized with p14 in Alum-based adjuvant. Pooled sera from mice inoculated with Alum only served as controls.

### Immunofluorescence (FITC)

Cells attached to polylysine-coated slides (Sigma, St. Louis, MO) were fixed (4% paraformaldehyde) and permeabilized (0.1% Triton X-100) prior to incubation with primary and fluorescent (FITC)-labeled secondary antibodies, respectively. Fluorescence was visualized in an IX70 fluorescent microscope (Olympus, Japan), equipped with a Coolsnap HQ digital camera (Photometrics, Tucson, AZ)

### Immunocytochemistry (Peroxidase)

pBC and MCF7 cell lines (expressing and lacking MMTV sequences, respectively) were plated on glass slides as a monolayer. The slides were fixed with Ethanol for 5 minutes and washed in PBS. For cell permeabilization, slides were dipped in Tween 20 - PBS (0,1% v/v) for 10 min and washed three times in PBS. Endogenous peroxidase was blocked by 3% hydrogen peroxide for 15 min. The slides were then incubated with primary antibody, (rabbit polyclonal anti-MMTV-p14 at 1:500 dilution) [29] for 1 h at room temperature, and for 30 min with biotinylated goat anti-rabbit IgG as secondary antibody (Invitrogen, Life Tecnologies, Grand Island, NY). ABC avidin/biotin reagent was added to the slides and incubated for 10 min. Slides were developed with diaminobenzidine chromogen (DAB) (DAKO, Glostrup, DK) and counterstained with hematoxylin. Negative controls included the omission of the primary antibody.

Slides were analyzed using an inverted microscope CARL ZEISS “Axio Observer Z1FLMot, and images were taken with CARL ZEISS “AXIOCAM Icc1” camera

### Adoptive cell transfer

*Splenocytes* from p14-His vaccinated, or naive mice were isolated according to standard procedure. *T- and B cells* were negatively selected using the EasySep mouse T or B cell isolation kits (Stem Cell Technologies, Vancouver, BC ) according to the manufacturer's protocol. Isolated cells were introduced IP into naïve mice followed an hour later by IP inoculation of T-67 cells. Mice were monitored daily.

### Monoclonal antibody treatment

Mice were inoculated IP with 1.5×10^6^ T-67 cells. This was followed by injecting a combination of anti-p14 monoclonal antibodies M-66 and M-202 (25μg each, in a total volume of 0.2ml, IP) every other day for 4 weeks. Control mice were inoculated with PBS. Mice were monitored daily.
